# Current Advances in Mosquito Bioecology and Control Strategies

**DOI:** 10.3390/insects17100995

**Published:** 2026-09-25

**Authors:** Athanasios Giatropoulos

**Affiliations:** Laboratory of Efficacy Control of Pesticides, Benaki Phytopathological Institute, 14561 Kifissia, Attica, Greece; a.giatropoulos@bpi.gr

Mosquitoes represent a major threat to both human and animal health due to serving as vectors for numerous serious diseases, including malaria, dengue fever, West Nile virus, and encephalitis. Factors such as climate change, urbanization, and the growth of global travel and trade have expanded the geographic distribution of invasive mosquito species and the diseases they transmit. Therefore, effective mosquito control requires not only an integrated mosquito management approach that combines all suitable control methods, but also a thorough understanding of the biology and ecology of the target mosquito species [1]. The current editorial article summarizes eleven research articles (herein referred as “Contributions”) collected for the second edition of the Special Issue entitled “Biology, Ecology and Management of Mosquitoes Affecting Humans and Animals”. This Special Issue presents recent advances in mosquito bioecology and control strategies, including the results from laboratory and field studies on mosquito fauna distribution, surveillance, insecticides, microbials, oviposition attractants, blood feeding behavior, and competitive interactions.

Yellow fever virus (YFV) is a mosquito-borne flavivirus maintained in sylvatic transmission cycles involving non-human primates and forest mosquitoes [2]. The study by Cardoso et al. (Contribution 1) investigated sylvatic Culicidae fauna and natural YFV infection in southern Santa Catarina, Brazil, following a recent outbreak. Between January and February 2023, 4352 female mosquitoes representing at least 32 species were collected in five municipalities. YFV screening by RT-LAMP detected natural infection in a pool of *Sabethes albiprivus*, representing the first record in southern Brazil and only the third worldwide. *Haemagogus leucocelaenus* was present in all sampling sites, whereas *Hg. janthinomys* was absent. These findings suggest a potential secondary role for *Sa. albiprivus* in YFV maintenance and reinforce the importance of entomological surveillance and vaccination for outbreak prevention.

Oviposition site selection is a key determinant of mosquito population dynamics and vector-borne disease transmission [3]. Agbetsi et al. (Contribution 2) investigated the role of indole—a microbial volatile associated with aquatic habitats—in oviposition behavior of gravid *Anopheles stephensi*. Water conditioned by first-instar larvae attracted females, whereas fourth-instar larval water was avoided, indicating discrimination between favorable and resource-depleted habitats. Furthermore, indole elicited strong oviposition preference across a broad concentration range (0.5–50 µM), with no clear dose-dependent response. Remarkably, antennae and maxillary palp removal did not abolish attraction to indole, suggesting that additional sensory structures contribute to cue detection. Expression of chemosensory genes in leg tissues supports a role for leg-mediated chemoreception in oviposition decisions, revealing previously underappreciated complexity in mosquito habitat assessment.

The increasing need for sustainable mosquito control has intensified interest in entomopathogenic fungi (EPF) as environmentally friendly alternatives to chemical insecticides [4]. The study by Mantzoukas et al. (Contribution 3) screened indigenous EPF isolated from forest soils in Achaia, Greece, for larvicidal activity against *Culex pipiens* biotype *molestus*, a vector of important arboviruses. Fifteen isolates belonging to *Beauveria* and *Metarhizium* species were evaluated. Significant differences in pathogenicity were observed, with *Beauveria bassiana* and *Metarhizium anisopliae* exhibiting the highest virulence, causing 60% larval mortality within 72 h and displaying rapid lethal action. Advanced survival and kinetic analyses confirmed distinct virulence profiles, highlighting the importance of local fungal biodiversity for developing effective biocontrol agents.

Understanding the blood feeding behavior of *Aedes aegypti* is essential for improving disease prevention strategies [5]. Ciomperlik et al. (Contribution 4) used a nanopore-based third-generation sequencing approach to identify blood-meal sources of *Ae. aegypti* collected in Houston, Texas, and Phoenix, Arizona. Blood-meal identification was successful for approximately 80% of specimens, revealing that most mosquitoes had fed on a single host, while about 20% showed evidence of multiple blood meals. Humans were the predominant host, followed by domestic cats and dogs. In Phoenix, mosquitoes also fed on birds, rodents, livestock, and even reptiles, demonstrating a broader host range than previously recognized. These findings highlight the value of advanced sequencing technologies for accurately characterizing mosquito host use and feeding complexity.

Pyriproxyfen (PPF), a juvenile hormone analog, is increasingly being explored as a complementary tool for malaria vector control and autodissemination strategies [6]. The study by Mnzava et al. (Contribution 5) examined how sublethal PPF exposure affects the fitness and insecticide susceptibility of pyrethroid-resistant *Anopheles arabiensis* over three generations. Mosquitoes exposed as larvae showed a temporary reduction in susceptibility to permethrin and deltamethrin during the first generation, but this effect was not inherited by subsequent generations. PPF exposure also imposed significant fitness costs, including reduced body size, lower egg production, and decreased egg hatchability. These negative effects on mosquito performance—combined with the absence of trans-generational resistance—suggest that PPF can weaken vector populations while maintaining the long-term effectiveness of insecticide-based interventions.

Microsporidia MB is a naturally occurring symbiont of *An. arabiensis* that prevents *Plasmodium* transmission without imposing measurable fitness costs on its mosquito host, making it a promising candidate for malaria control [7]. Boanyah et al. (Contribution 6) investigated the transgenerational dynamics of Microsporidia MB under semi-field conditions over six mosquito generations. Populations established from naturally infected females were monitored for symbiont prevalence and infection intensity using qPCR. Microsporidia MB prevalence and intensity increased during the early generations (F1–F4) before declining in later generations. A weak positive association was observed between prevalence and temperature, while humidity showed no clear effect. Females exhibited higher infection intensities than males, whereas male body size remained stable across generations. These findings improve our understanding of Microsporidia MB persistence and support its potential application in symbiont-based malaria control strategies.

Larval crowding is an important ecological factor influencing mosquito development and adult morphology [8]. In the study by Aytekin et al. (Contribution 7), the authors examined the effects of increasing larval density on the wing size and shape of *Cx. pipiens*, using landmark-based geometric morphometrics and Elliptic Fourier Analysis. Both approaches revealed significant density-dependent morphological changes, with wing size progressively decreasing as larval density increased. A critical threshold was identified at approximately 1 larva/mL, beyond which consistent wing deformations became evident in both sexes. Changes affected not only wing contours but also wing vein architecture, indicating that larval density independently influences morphological development. These findings highlight the importance of environmental stressors in shaping mosquito phenotypes and suggest that density-induced variation should be considered in taxonomic, ecological, and evolutionary studies.

Oviposition attractants are valuable tools for improving the surveillance and control of disease-transmitting *Aedes* mosquitoes [3]. Xie et al. (Contribution 8) evaluated teas at different fermentation stages as potential botanical attractants for gravid *Aedes albopictus* and *Ae. aegypti*. Among the teas tested, post-fermented Liu-pao tea consistently showed the strongest attraction, particularly at 5 g/L after 7–14 days of infusion. Even low-concentration infusions attracted *Ae. aegypti* after extended aging. Autoclaving did not reduce attractiveness, indicating that volatile plant-derived compounds, rather than live microorganisms, drive mosquito responses. Chemical analyses identified cedrol as a contributing attractant, although whole-tea infusions were considerably more effective. These findings highlight Liu-pao tea as a promising source of natural oviposition attractants for environmentally friendly mosquito management strategies.

The Amazon rainforest harbors an exceptional diversity of mosquitoes, many of which are important vectors of human and animal pathogens [9]. The study by Nascimento et al. (Contribution 9) characterized the Culicidae fauna of Mazagão, Amapá, a poorly surveyed region of the eastern Brazilian Amazon. Mosquitoes were sampled during rainy, intermediary, and dry seasons using protected human attraction and CDC light traps positioned at ground and canopy levels. A total of 3500 specimens representing 38 species and 15 genera were collected. Species richness and abundance peaked during the intermediary season, whereas severe drought and forest fires were associated with markedly reduced diversity during the dry period. The genera *Culex*, *Coquillettidia*, and *Mansonia* predominated, including species of epidemiological importance. These findings expand knowledge of Amazonian mosquito biodiversity and highlight the influence of seasonality and environmental disturbance on mosquito community structure.

*Aedes albopictus* and *Ae. aegypti* are major vectors of dengue and chikungunya and often coexist in urban and peri-urban environments, complicating vector control strategies [10]. Vitry et al. (Contribution 10) evaluated larval habitat sharing and trap efficiency in Saint-Joseph, La Réunion, in preparation for a boosted sterile insect technique program. Two adult trap types were compared under a Latin square design using CO_2_ bait with and without attractants, alongside surveys of natural breeding sites. CO_2_-baited BG Sentinel traps effectively captured both species, while ovi-sticky traps performed poorly. Larval surveys showed *Ae. aegypti* occupying a limited number of *Pandanus utilis* habitats, whereas *Ae. albopictus* was more widespread, with evidence of negative larval co-occurrence in shared sites. These results support optimized CO_2_-based trapping for monitoring and confirm overlapping but ecologically differentiated breeding patterns.

Citizen science in mosquito surveillance engages the public to report and share mosquito data via apps, expanding monitoring coverage [11]. Eritja et al. (Contribution 11) assessed a dual surveillance strategy in Spain combining conventional entomological field sampling with citizen science data generated through the Mosquito Alert platform. Municipal-level detections from 2004 to 2024 were analyzed to evaluate long-term invasion dynamics. Field surveillance ensured high diagnostic reliability, while citizen science contributed large-scale, real-time reporting and accounted for a substantial proportion of detections since 2014. The integration of mobile reporting, AI-assisted classification, and expert validation improved both spatial resolution and early detection capacity, particularly for long-distance dispersal events. The results demonstrate that combining participatory monitoring with classical entomology enhances the effectiveness of early-warning systems for invasive mosquito species.

Overall, the following summarizes the primary findings of the research articles included here:

- In southern Brazil, the first natural YFV infection in *Sabethes albiprivus* was documented, and *Haemagogus leucocelaenus* was identified as the most probable primary vector sustaining yellow fever transmission in the region (Contribution 1);
- Gravid *An. stephensi* females retained a strong preference for indole-containing oviposition sites even after antennal and maxillary palp ablation, indicating that alternative sensory organs—likely the legs—contribute to indole detection and oviposition-site selection (Contribution 2);
- Two indigenous fungal isolates, *Beauveria bassiana* and *Metarhizium anisopliae*, were identified as highly virulent against *Cx. pipiens* larvae, demonstrating strong potential as environmentally sustainable mosquito biocontrol agents (Contribution 3);
- Third-generation nanopore sequencing revealed that *Ae. aegypti* feeds primarily on humans and domestic animals but can exploit a much broader range of hosts, while also enabling the accurate detection of multiple blood meals within individual mosquitoes (Contribution 4);
- Sublethal exposure to pyriproxyfen temporarily altered pyrethroid susceptibility and significantly reduced the reproductive fitness of *An. arabiensis*, but these effects were not inherited by subsequent generations (Contribution 5);
- Microsporidia MB successfully persisted and spread across multiple generations of *An. arabiensis* under semi-field conditions, although its prevalence and infection intensity declined after an initial increase, highlighting the need to better understand factors influencing long-term symbiont stability (Contribution 6);
- High larval density caused progressive reductions in wing size and significant wing shape deformations in *Cx. pipiens*, with a clear morphological breaking point occurring at approximately 1 larva/mL (Contribution 7);
- Post-fermented Liu-pao tea was identified as a highly effective botanical oviposition attractant for *Aedes* mosquitoes, with its activity driven primarily by tea-derived volatile compounds rather than microbial processes (Contribution 8);
- A mosquito survey in Mazagão, eastern Amazon, revealed a diverse mosquito fauna with strong seasonal variation in species composition and abundance, emphasizing the potential public health importance of continuous entomological monitoring (Contribution 9);
- CO_2_-baited BG Sentinel traps were the most effective for monitoring both *Ae. albopictus* and *Ae. aegypti*, and the two species showed partially overlapping but ecologically differentiated larval habitats in La Réunion (Contribution 10);
- Combining citizen science (Mosquito Alert platform) with traditional entomological surveillance significantly enhanced the early detection, spatial coverage, and long-term monitoring of invasive *Aedes* mosquitoes in Spain (Contribution 11).
